# The impact of COVID‐19 on patients with neurological disorders and their access to healthcare in Africa: A review of the literature

**DOI:** 10.1002/brb3.2742

**Published:** 2022-08-11

**Authors:** Olivier Uwishema, Kristian Steen Frederiksen, Inês F. Silva Correia, Ashraf Mahmoud, Helen Onyeaka, Burhan Dost

**Affiliations:** ^1^ Oli Health Magazine Organization Research and Education Kigali Rwanda; ^2^ Department of Projects and Research Clinton Global Initiative University New York City New York USA; ^3^ Faculty of Medicine Karadeniz Technical University Trabzon Turkey; ^4^ Danish Dementia Research Centre Copenhagen Danmark; ^5^ School of Medicine, Faculty of Health, Education, Medicine & Social Care Anglia Ruskin University Chelmsford UK; ^6^ Faculty of Medicine Kilimanjaro Christian Medical University College Moshi Tanzania; ^7^ School of Chemical Engineering University of Birmingham Edgbaston UK; ^8^ Department of Anaesthesiology, School of Medicine Ondokuz Mayis University Kurupelit Turkey

**Keywords:** Africa, COVID‐19, healthcare, neurological disorders

## Abstract

**Introduction:**

The coronavirus disease 2019 (COVID‐19) pandemic has hampered the progress of neurological healthcare services for patients across Africa. Before the pandemic, access to these services was already limited due to elevated treatment costs among uninsured individuals, shortage of medicines, equipment, and qualified personnel, immense distance between residing areas and neurological facilities, and a limited understanding of neurological diseases and their presentation by both the health workers and the African population.

**Methodology:**

The databases PubMed, Google Scholar, Science Direct, and the National Library of Medicine were searched for literature. All articles on neurological disorders in Africa were considered.

**Aim:**

This review article explores the challenges of providing the best services for patients suffering from neurological disorders in Africa amid the COVID‐19 pandemic and provides evidence‐based recommendations.

**Results:**

As Africa's governments made more resources available to support patients affected by COVID‐19, neurological care received less priority and the capacity and competency to treat patients with neurological disorders thus suffered substantially. Both short‐term and long‐term strategies are needed to improve the quality of neurological services after the pandemic in the region.

**Conclusion:**

To strengthen Africa's neurological services capability during and after the COVID‐19 pandemic, African governments must ensure appropriate healthcare resource allocation, perform neurology management training, and increase health security measures in medication supply. Long‐term strategies include incorporating responsible finance and resource procurement and advancement of tele‐neurology. International collaboration is essential to promote the sustainable improvement of neurological services in Africa.

## 1 BACKGROUND

Since the beginning of March 2020, when the World Health Organization (WHO) declared the outbreak of the new coronavirus disease 2019 (COVID‐19) as a pandemic (WHO, 2020a), nations worldwide have fought tirelessly to control the spread of the disease. As the disease's prevalence increased, healthcare systems redirected all the efforts to care for patients infected with COVID‐19, especially in intensive care units (ICU), while other departments saw a depletion in their resources and staff, especially those with neurological disorders and pre‐existing chronic health conditions (Bhaskar, Bradley, et al., 2020; Uwishema, Berjaoui, et al., 2022; Minni et al., 2020; Robertson et al., 2020) as well as acute facilities accessed by many patients (Bhaskar, Sharma, et al., 2020; Siegler et al., 2020).

COVID‐19 has severely affected emerging nations as they had to bear an additional burden as a result of this pandemic (Fortuna et al., 2020). In the African Region, this has led to a major disruption of mental, neurological, and substance services’ usage (54%) compared to high‐income countries (24%) (WHO, 2020b). Furthermore, the pandemic has compelled many healthcare‐providing facilities to use telemedicine as an alternative to in‐person care of chronic patients (Hollander & Carr, 2020); a scenario that poses a challenge to accessing healthcare among the African population. On one hand, a technological transformation of this magnitude requires a developed infrastructure, including robust and fast internet access and training of healthcare workers, both of which are inadequate in Africa, due to the continent's slow financial and technological development (Ajakaiye & Ncube, 2010). This shift of technology‐related healthcare provision is less likely to be successful in remote settings, such as in rural communities, where 60% of the African population inhabits and where access to technology is scarce, compared to the available outpatient services in hospitals (Internet World Stats, 2019; UNFPA, 2019). As the continent faces the pandemic crisis, technological and health services improvement at the moment will be challenging to the countries that struggle economically (Patel et al., 2020).

On another hand, there are only 0.043 neurologists per 100,000 people in the WHO African Region, compared to 4.84 neurologists per 100,000 people in Europe (Musubire et al., 2021). This shortage of specialists reduces the ability to provide effective care to patients. First, since attending a medical facility could increase the risk of contracting the SARS‐CoV‐2 virus, patients are more likely to cancel their regular follow‐ups, leaving people with neurological disorders more fragile and prone to more devastating complications (Cuffaro et al., 2020). Secondly, the treatment of many neurological conditions, such as multiple sclerosis and autoimmune neuropathies, consists of immunosuppressive medication, which can predispose patients to contracting COVID‐19 and presenting with severe complications (Manji et al., 2020; Willis & Robertson, 2020). Third, patients with neuromuscular diseases can suffer exacerbations of their condition including respiratory distress upon catching an infection (Guidon & Amato, 2020; Gummi et al., 2019).

To enhance the health and quality of life of patients with neurological disorders in Africa, it is critical to examine the state of care before the pandemic, taking into account the present state of neurological disorders, neurological services and giving the best care possible to minimize additional risks which is the aim of this article.

## 2 ACCESSIBILITY OF HEALTHCARE SERVICES FOR PATIENTS WITH NEUROLOGICAL DISORDERS IN AFRICA BEFORE THE COVID‐19 PANDEMIC

Accessibility to healthcare services comprises four main pillars, namely healthcare coverage, services availability, timeline for accessing care, and lastly the workforce involved in managing or treating diseases (AHRQ, 2018).

To begin with, the difficulties faced by neurological care are caused by the lack of facilities and insurance coverage which is also exacerbated by inefficiently operating equipment (Sarfo et al., 2017). The national healthcare system of different African countries offers health insurance that covers a few of the fundamental tests, such as complete blood count and human immunodeficiency virus (HIV) tests (Diaz, 2019), which are an important element of most clinicians’ diagnostic armamentarium. However, patients must pay hefty prices for some neurological tests (e.g., brain scans) and hospital stays (Diaz, 2019). Even if they are accessible, most of these imaging technologies do not function properly, forcing patients to seek private care at a greater cost, increasing their financial burden and challenging the healthcare coverage in terms of neurological care (Diaz, 2019).

What remains more concerning is that the treatment gap, defined as “the difference between patients with active disease who receive treatment and patients with active disease who do not” (Kohn et al., 2004), is common in neurological diseases in developing countries with “the treatment gap for epilepsy being more than 75% in low‐income counties and 50% in moderate‐income counties, compared to less than 10% in developed countries” (Meyer et al., 2010). The disparity in medicines and services availability widens when, even though hospitals supply some free medication, the majority of neurological drug treatment is either unaffordable or unavailable (Diaz, 2019; Mbuba et al., 2012; Williams et al., 2018).

Moreover, neurological therapy is often limited to tertiary centers and teaching hospitals, which can be troublesome for patients in rural areas who must travel considerable distances to reach a healthcare facility (Mbuba et al., 2012). Because the majority of neurological patients reside in places where knowledge about health and the body is considerably low (Awad Bashir & Cumber, 2019; Institute of Medicine (US) Forum on Neuroscience and Nervous System Disorders and Uganda National Academy of Sciences Forum on Health and Nutrition, 2010), they are frequently subjected to societal and cultural stigma by being neglected and isolated. As their symptoms are frequently explained by traditional beliefs in their communities, such as behavioral changes or seizures, they seek traditional healers rather than medical help (Akinyemi et al., 2021; Mbuba et al., 2012), which disrupts healthcare provision from the provider level to the client level, resulting in lower adherence to healthcare‐seeking behavior (Institute of Medicine (US) Forum on Neuroscience and Nervous System Disorders and Uganda National Academy of Sciences Forum on Health and Nutrition, 2010).

A country's spoken and written language can also have a profound impact on patient care. Language may be a barrier between patients and physicians in a speciality like neurology, where history taking is a cornerstone of the diagnostic workup, considering the African population speaks up to 2100 distinct languages (Diaz, 2019; Hutchison et al., 2001). Moreover, having one neurologist for every 2,300,000 people (0.043/100,000) in the African Region, where as the WHO recommending at least one for every 100,000 inhabitants questions the quality, competency, and the capability to provide neurological services to patients (Musubire et al., 2021; World Health Organization, 2017). Other reasons for the scarcity of neurological health services include a lack of or a shortening of training, limited resources for proper diagnosis, a shortage of specialized neurological nurses, neurologists migrating for better training and work environment, and a diagnosis based primarily on history and examination with no confirmation by imaging and other tests (Diaz, 2019; Awad Bashir & Cumber, 2019; Williams et al., 2018).

## 3 THE BURDEN OF NEUROLOGICAL DISEASE AND THE CURRENT STATUS OF NEUROLOGICAL SERVICES IN AFRICA DURING THE COVID‐19 PANDEMIC

According to a WHO assessment, 71% of African nations have seen disruptions in chronic disease care, including noncommunicable diseases (NCD) (WHO, 2020b). Although the majority of African governments made an oath in 2001 to allocate more funds and resources to combat NCD, the majority of African countries do not have a budget for NCDs healthcare programs (Jerving, 2018). The lack of resources has had an immediate and indirect impact on neurological services since different NCDs, including hypertension and diabetes, whose healthcare programs were also disrupted during the pandemic, are major risk factors for stroke and other neurological diseases (Leone et al., 2021; Meyer et al., 2010; Uwishema, Ayoub, et al., 2022).

According to reports, an interruption in HIV treatment for up to 6 months will result in 500,000 more deaths from AIDS‐related diseases, and a lack of mother‐to‐child transmission prevention, increasing the incidence rates of new infections. The most serious healthcare concern is that HIV, even with an undetectable viral load, has been linked to stroke and epilepsy in the Sub‐Saharan region (Benjamin et al., 2016; Mateen et al., 2012; Uwishema, Onyeaka, et al., 2022). As a result, combined preventive strategies in general care and antiretroviral treatment (ART) facilities have been proposed (Uwishema, Ayoub, et al., 2022; UNAIDS, 2020a, 2020b) and, with increasing evidence of stroke and epilepsy cases among HIV patients, the United Nations has proposed a unified management program (UN, 2011), which has, regrettably, faced numerous obstacles that were exacerbated during the COVID‐19 pandemic:

- A dearth of staffing: The lack of skilled and qualified personnel to treat neurological problems in Africa as well as investigate improvements in the healthcare sector (Musubire et al., 2021; Søvold et al., 2021).
- Continued treatment: Patient compliance is critical to the effectiveness of chronic disease therapy. However, due to infrequent ART center visits, the pandemic has led to low patient compliance and adherence to medications (Ahmed et al., 2022).
- Poverty: The difficulties that patients encounter while paying for medical care due to limited financial resources and a lack of healthcare insurance facilities (Xu et al., 2003).

African countries have also adopted telehealth/teleconsultation frameworks in response to the ongoing pandemic. Teleneurology is a developing discipline of telemedicine in which neurological counselling is provided at a distance or remotely using different technologies, including the internet, continuous mobile healthcare, and remote epilepsy therapy (Kuchenbuch et al., 2020; Leone et al., 2018; Larner, 2011). Although already adopted in several African nations before the pandemic, as part of The Disease Relief through Excellent and Advanced Means program, these services have been shown to improve during and after the COVID‐19 to meet the shortage of neurological health care in Africa (Adebayo et al., 2021). Teleneurology in Africa is still in its early stages, but it is the most potent initiative to date, being both cost‐effective and accessible to a larger population.

Hospital admissions and elective procedures were also compromised by the necessity to support patients with COVID‐19, which resulted in a reduction in the availability of hospital beds, in medical wards and ICU (Ciarleglio et al., 2021). Emergency neurological services were also affected, proven by a decrease in stroke and transient ischemic attacks patients, as well as the number of patients who came to emergency departments with acute and refractory headaches (Burlica, 2021). Another challenge posed by the ongoing pandemic is the impact on the continent's medicine supply chains as a result of closed borders, lockdowns, and movement restrictions. This decrease in supply has raised medication costs, leading to a rise in black‐market sales of counterfeit, low‐quality, and ineffective drugs (Uwishema, Mahmoud, et al., 2022; UNAIDS, 2020a). These counterfeit drugs have been reported to have an impact on the care of stroke and epilepsy patients. For example, a quality check on five antihypertensive drugs used in stroke has revealed that they are substandard, with potentially negative consequences in ten Sub‐Saharan African countries (Macquart De Terline et al., 2018). Another case in point was a counterfeit antiepileptic drug that caused rapid loss of seizure control in 63%–88% of the population, with two deaths attributable to false phenobarbital tablets (Otte et al., 2015).

Although it is not statistically clear how COVID‐19 has exacerbated neurological morbidity in Africa due to the lack of data and surveillance programs, WHO expects the pandemic to cause an increase in mortality due to the disruption of health services, particularly for patients who require regular or long‐term neurological care (WHO, 2020b).

## 4 RECOMMENDATIONS

The dismal state of neurological services in Africa emphasizes the need for systemic improvements in healthcare delivery. Following the WHO Atlas project for neurological services (World Health Organization 2017) that includes 34 African countries, we have considered certain recommendations to assess and alleviate neurological care during a pandemic as well as in the long term:

1. Policies in neuro‐disorders: As stated by the Atlas, a total of 24% of countries across the world report neurological health policies with a bigger deficit in low‐ and middle‐income countries (World Health Organization, 2017). Conducting public healthcare awareness campaigns to boost Africans’ trust in healthcare as well as their understanding of these policies (Diaz, 2019) will improve their willingness to seek medical attention. With visionary neurological health policies, the quality of health‐care delivery, accessible health‐service and engagement of people with neurological conditions and their families will improve drastically in the African continent (World Health Organization, 2017).
2. Legislation for neuro‐disorders: Neurological healthcare legislation needs to be focused on issues that protect patients human rights, for instance working environment restrictions and protections training and services structure in Africa (World Health Organization, 2017). Legislations are crucial indicators for good governance, for example, legislations on neurological disorders may include medical aptitude to drive in people with epilepsy or the ability to act in people with dementia or other neurological disorders (World Health Organization, 2017). Such legislations will not only improve living conditions of people with neurological conditions they will also improve the care or service provided.
3. Financing for neuro‐disorders: Strengthen the region's capacity for neurological services by implementing efficient healthcare system finance (Adebayo et al., 2021). Furthermore, access should be improved by developing a cost‐effective healthcare delivery model to pave the path for accessible teleneurology services (Adebayo et al., 2021). Although it is a challenging goal, it would be essential to assess the government's budget for neurological services as well as utilize the support from more developed countries (World Health Organization, 2017).
4. Social welfare support for neuro‐disorders: Identify gaps and strengthen health security (the presence of strong and resilient public health systems in the community) in the present African healthcare supply chain (CDC, 2021; Gebremeskel et al., 2021). Moreover, assistance for individuals and their families who suffer from neurological disorders should be assessed and the government should seek support from more wealthy nations.
5. Workforce for neuro‐disorders: Deliver neurology emergency management training to nonspecialized healthcare personnel in response to the region's scarcity of neurology experts (Adebayo et al., 2021). Furthermore, with the ratio of 3.1 per 100,000 population of the total neurological workforce, more extensive training should be put in place (World Health Organization, 2017).
6. Services for neuro‐disorders: Allocate more healthcare resources and personnel designated for neurological services, primarily to those departments responsible for the treatment of emergency cases (Otte et al., 2015). In Africa, the disparity between adult neurologist, children neurologists, and neurosurgeons is quite huge and shows there is a need to refocus efforts to train competent and qualified personnel to deal with neurological challenges within the continent (World Health Organization, 2017).
7. Information gathering systems neuro‐disorders: It has been reported that close to 42% of world countries do not retain any neurological disorder data to be studied by researchers and clinicians. To improve healthcare services, more research is required with the support of world‐leading research centers and accessible technology (World Health Organization, 2017).
8. Neuro‐professional associations and nongovernmental organizations: In executing the aforementioned strategies, nongovernmental organizations and international bodies should be actively involved by providing expert assistance and ensuring accountability programs. It is also essential to strengthen pan‐African collaboration through technology transfer and knowledge exchange about neurology and teleneurology (Adebayo et al., 2021).

## 5 CONCLUSION

Worldwide, neurological disorders are among the leading causes of morbidity and mortality. Even before the COVID‐19 pandemic, securing access to neurological services in Africa was problematic due to the persistent shortage of medical supplies and healthcare workers. To strengthen Africa's neurological services capability during and after the pandemic, African governments must ensure appropriate healthcare resource allocation, perform neurology management training, and increase health security measures in medication supply. Long‐term strategies include incorporating responsible finance and resource procurement, advancement of teleneurology, and international collaboration are essential to promote the sustainable improvement of neurological services in Africa.

## CONFLICTS OF INTEREST

The authors declare no conflict of interest.

## FUNDING INFORMATION

None.

## AUTHOR CONTRIBUTIONS

Olivier Uwishema was involved in conceptualization, project administration, writing‐review and designing, and reviewing and editing of the first draft. Kristian Steen Frederiksen supervised, reviewed the second draft, edited and made critical comments. Helen Onyeaka reviewed and edited the second draft. Inês F. Silva Correia reviewed and edited the third draft. Burhan Dost reviewed and edited the final draft. All authors were involved in collection and assembly of data, data analysis and interpretation, manuscript writing, and final approval of manuscript.

### PEER REVIEW

The peer review history for this article is available at https://publons.com/publon/10.1002/brb3.2742

## Data Availability

Not Applicable
